# Deep Convolutional Neural Network-Based Early Automated Detection of Diabetic Retinopathy Using Fundus Image

**DOI:** 10.3390/molecules22122054

**Published:** 2017-11-23

**Authors:** Kele Xu, Dawei Feng, Haibo Mi

**Affiliations:** 1 School of Information and Communication, National University of Defense Technology, Wuhan 430019, China; kelele.xu@gmail.com; 2 School of Computer, National University of Defense Technology, Changsha 410073, China; haibo_mihb@126.com

**Keywords:** diabetic retinopathy, deep convolutional neural network, image classification

## Abstract

The automatic detection of diabetic retinopathy is of vital importance, as it is the main cause of irreversible vision loss in the working-age population in the developed world. The early detection of diabetic retinopathy occurrence can be very helpful for clinical treatment; although several different feature extraction approaches have been proposed, the classification task for retinal images is still tedious even for those trained clinicians. Recently, deep convolutional neural networks have manifested superior performance in image classification compared to previous handcrafted feature-based image classification methods. Thus, in this paper, we explored the use of deep convolutional neural network methodology for the automatic classification of diabetic retinopathy using color fundus image, and obtained an accuracy of 94.5% on our dataset, outperforming the results obtained by using classical approaches.

## 1. Introduction

**Figure 1:**
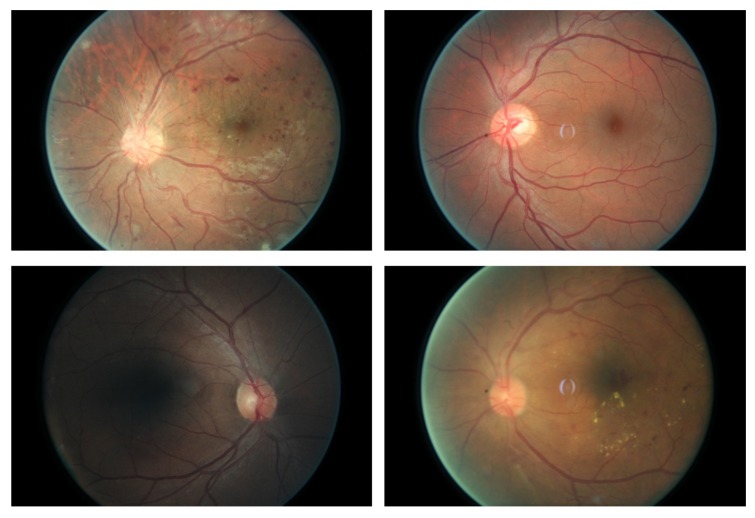
Sample frames of the retina images. The first two frames in the top row come from normal subjects, while the two frames in the bottom row come from the patients who have diabetic retinopathy.

The number of people diagnosed as having diabetes has increased dramatically over the last several decades, and diabetes increases the risk of a range of eye diseases, of which diabetic retinopathy is one of the most severe [1]. Moreover, diabetic retinopathy is the main cause of blindness in the mid-age population [2]. Despite sustained efforts having been made, early detection of diabetic retinopathy is a time-consuming process even for a well–trained clinician, which may result in delayed treatment, miscommunication, etc. The importance of an automatic method for diabetic retinopathy detection has been recognized. In our study, we focus on the classification of retinal images into normal images and diabetic retinopathy images (sample frames used for our classification problem are given in Figure 1). Previous efforts using image feature extraction and machine learning methods made good progress. The features used for the classifiers include hard exudates [3,4], red lesions [5], micro-aneurysms and blood vessel detection [6], etc., while the classifiers used for the task contain neural networks, sparse representation classifiers, linear discriminant analysis (LDA), support vector machine (SVM), k-nearest neighbors (KNN) algorithm and so on. However, none of the handcrafted features can cover all the symptoms of diabetic retinopathy in the images, and a large fraction of cases turn out to be normal while much time has been spent diagnosing normal cases. Consequently, the practical clinical applications of the automatic diagnosing system are limited.

Recent advances in convolutional neural networks (CNNs) have made it a state-of-the-art technique in image classification tasks [7], and its variants have begun to dominate many fields in computer vision, such as object detection [8], image classification [9], object tracking [10], edge detection [11]. Instead of making essential use of handcrafted features, CNN can learn a hierarchy of features, which can be used for image classification purposes. As the hierarchy approach is available to learn more complex features, as well as translation and distortion features in higher layers, the accuracy of the CNN-based image classification method can be higher. Based on this assumption, we explore the use of the CNN-based method for the diabetic retinopathy test in this work. Moreover, a specific multi-layer CNN architecture is designed, and experiments are conducted on real retina data. In addition, the results obtained demonstrate our assumption as we achieve 94.5% accuracy, which ranks as the highest in comparison with previous handcrafted feature-based classifiers. This paper is organized as follows: Section 2 describes the preprocessing method and gives an overview of the CNN architecture used in this work, while Section 3 presents the results to demonstrate the performance of the proposed approach compared to previous handcrafted feature-based classification methods. The conclusion and future work are discussed in Section 4.

## 2. Methodology

### 2.1. Data Augmentation

**Figure 2:**
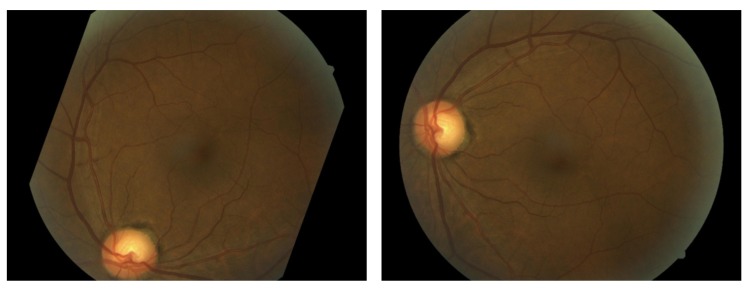
Samples of the transformed frames.

Until recently, datasets of labeled images with diabetic retinopathy were relatively small. Here, we used the data provided by Kaggle Community [12]. Indeed, the shortcomings of small image datasets have been widely recognized, thus, data augmentation is needed to artificially enlarge the datasets using label-preserving transformation, which can reduce overfitting on the image data and increase the performance of the algorithm [13]. In our experiment, we apply translation, stretching, rotation and flipping to the labeled dataset. A summary of the transformations is given in Table 1, while the sample transformed frames are presented in Figure 2. Five different transformation types are used in our experiment, including rotation, flipping, shearing, rescaling and translation. For each type, the parameter details are given in Table 1.

### 2.2. Convolutional Neural Network-Based Image Classification

The convolutional neural network (CNN) belongs to the feed-forward artificial neural network (ANN), which is very similar to ordinary neural networks. The CNN is a well-known deep learning architecture, in which individual neurons are tiled in such a way that they respond to overlapping regions in the visual fields [14]. CNNs are an important class of learnable representation applications, and they were inspired by biological neural networks. Numerous variants have been proposed over last several years. However, the basic components are very similar. CNNs consist of alternating convolution and pooling operations [15]. Typically, the convolutional layers are interspersed with pooling layers to reduce computation time, and build up further spatial and configuration invariance [16]; the last few layers (close to the outputs) will be fully connected 1-dimensional layers. In more detail, a feed-forward neural network can be viewed as a function *f* of mapping data *x*:

(1) $$f\left(x\right)=f_{L}\left(\ldots f_{2}\left(f_{1}\left(x_{1},w_{1}\right),w_{2}\right)\ldots ,w_{L}\right).$$

**Figure 3:**
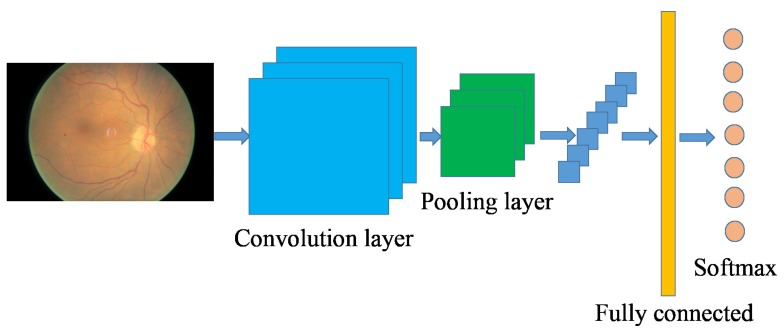
An exemplary architecture of the convolutional neural network.

Each function $f_{l}$ takes $x_{l}$ ($x_{1}$ is the input data *x*) as input with a learnable parameter vector $w_{l}$. *L* denotes the depth of the neural network. Although the type and sequence of functions are usually handcrafted, the parameters can be discriminatively learned from example data such that the resulting function f realizes a useful mapping. Formally, in a CNN, each $x_{l}$ will be a *M*×*N*×*C* array. As our problem can be simplified as a binary-classification problem, we can define the loss function of the CNN as:

(2) $$L\left(w\right)=1/n\times (\sum_{i=1}^{n}l\left(z_{i},f\left(x_{i};w\right)\right)),$$

where *n* is the number of samples, $z_{i}$ is the true label of sample *i*. The training problem can be converted to training a neural network to minimize the loss function *L*. Figure 3 gives the general architecture of a CNN network, which consists of multiple layers of small neurons. The results of these collections are then tiled so that they overlap to obtain a better representation of the original image (such as edges in the image). Convolutional layers consist of a rectangle grid of neurons, which takes a rectangle region of the previous layer as input. Moreover, there may be several grids in each convolutional layer, using potentially different filters. Typically, there is a pooling layer after each convolutional layer, which are subsampled from the previous convolutional layer. This pooling can be carried out in several ways, such as the average, maximum, etc. Finally, after several convolutional layers and max pooling layers, a fully connected layer (or several layers) will be built using outputs from previous layers (maybe the fully connected, pooling or convolutional layer), which is used as a compact feature to describe the whole input image. The network is optimized by backpropagation and stochastic gradient descent. Note that the forward and backward propagations may differ depending on the type of the layer.

Several different CNN architectures have been proposed and evaluated in our experiments. The depth of the tested neural network ranges from 9–18, and the convolution kernel size ranges from 1 to 5. To fit the input size of the CNN, we resize the image size to 224 × 224 × 3. The final architecture of the network used in our work is given in Table 2. For a given input, the network outputs two probabilities that sum up to 1, one for each class (our problem is a binary classification problem). In our experiment, 800 labeled images are used to train the neural network, while 200 images are used to evaluate the performance of the trained neural network.

## 3. Experimental Results

To evaluate the performance of the proposed method, the classification task was conducted using the CNN and Gradient boosting machines. Moreover, to compare the results obtained by automatic classification algorithms with the performance of human judgement, a human specialist is introduced to label the images as ground-truth.

**Figure 4:**
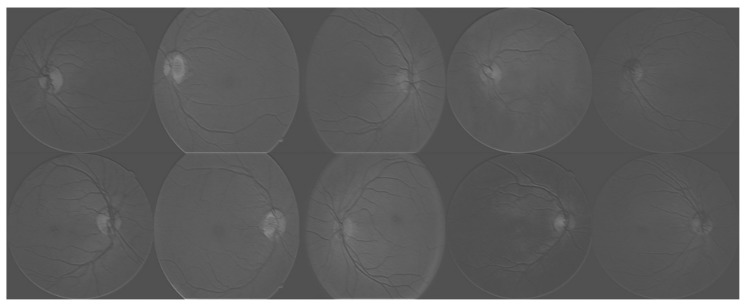
Visualization of the trained neural networks. Each image represents the activations of the first layer during the forward pass.

In more detail, four different feature extraction approaches have been employed: hard exudates, red lesions, micro-aneurysms and blood vessel detection. Two different kinds of classifiers were trained for the classification task: one kind of which combines the aforementioned extracted features and gradient boosting trees-based (GBM) classification method (Hard exudates + GBM, Red lesions + GBM, Micro-aneurysms + GBM and Blood vessel detection + GBM shown in Table 3.), and the other kind is the CNN-based methods (with or without data augmentation). Specifically, we use the default hyper parameters for GBM, with the number of classes set to 2, and the maximum depth set to 6. The GBM package used in this paper is the eXtreme Gradient Boosting method (XGBoost) [17], as it shows superior performance in our experiments when compared to other approaches (i.e., Support Vector Machine, Random Forest). Regarding implementation of the CNN, we made use of the R package named “MXNet” [18]. A visualization of the trained neural networks is given in Figure 4.

The classification accuracies are given in Table 3. As can be seen from the table, the CNN-based method provides superior performance compared to other methods, which supports the assumption made in the introduction section. Also, as can be seen from the table, the results obtained using the CNN with data augmentation are better than the CNN without data augmentation, and the reason may be that the data augmentation can be helpful for the CNN to deal with small rotation or translations during the data recording.

Our experiment was conducted on a Windows 8 operation system with Intel 4-Core 3.7GHz CPU, 16GB RAM, Dual AMD Filepro 512GB PCIe-based flash storage, and a Geforce 1070GPU. Although the training process of the CNN required 2 days, the trained network can provide the probability of diabetic retinopathy in less than 1 second, which can be used by clinicians in practice [19].

## 4. Discussion and Conclusions

With a limited number of medical staff, an automated system can significantly decrease the tedious manual labor involved in diagnosing large quantities of retinal images. Feature extraction-based diabetic retinopathy diagnosis has played a dominant role in previous studies. However, the progress made in deep convolutional neural networks has led to them becoming a state-of-the-art technique in optical image classification. In this paper, we explored the potential usage of the CNN in retinal image classification. The contribution of this paper is two-fold: firstly, we proposed a special neural network architecture for the diabetic retinopathy image classification task, which demonstrates superior performance over conventional feature extraction-based methods. Moreover, a data augmentation method was introduced for the proposed algorithm, which also improves the algorithm’s performance. The results are encouraging compared to the reports of human grading [20], thus a clinical evaluation will be undertaken in order to be able to integrate the presented algorithm into a tool to diagnose diabetic retinopathy [21].

## Figures and Tables

**Table 1:** Data augmentation parameters.

Transformation Type	Description
Rotation	0${}^{\circ }$–360${}^{\circ }$
Flipping	0 (without flipping) or 1 (with flipping)
Shearing	Randomly with angle between −15${}^{\circ }$ and 15${}^{\circ }$
Rescaling	Randomly with scale factor between 1/1.6 and 1.6
Translation	Randomly with shift between −10 and 10 pixels

**Table 2:** Convolutional neural network (CNN) architecture used in our experiment.

Output Shape	Description
224 × 224 × 3	input
222 × 222 × 32	3 × 3 convolution, 32 filter
220 × 220 × 32	3 × 3 convolution, 32 filter
110 × 110 × 32	2 × 2 max-pooling
108 × 108 × 64	3 × 3 convolution, 64 filter
106 × 106 × 64	3 × 3 convolution, 64 filter
53 × 53 × 64	2 × 2 max-pooling
53 × 53 × 128	3 × 3 convolution, 128 filter
51 × 51 × 128	3 × 3 convolution, 128 filter
49 × 49 × 128	2 × 2 max-pooling
24 × 24 × 256	3 × 3 convolution, 256 filter
22 × 22 × 256	3 × 3 convolution, 256 filter
11 × 11 × 256	2 × 2 max-pooling
4096	flatterned and fully connected
1024	fully connected
2	softmax

**Table 3:** Performance comparison with different approaches.

Method	Accuracy
Hard exudates + GBM	89.4%
Red lesions + GBM	88.7%
Micro-aneurysms + GBM	86.2%
Blood vessel detection + GBM	79.1%
CNN without data augmentation	91.5%
CNN with data augmentation	94.5%
